# Mutual connected IL-6, EGFR and LIN28/Let7-related mechanisms modulate PD-L1 and IGF upregulation in HNSCC using immunotherapy

**DOI:** 10.3389/fonc.2023.1140133

**Published:** 2023-04-12

**Authors:** Junjun Li, Yazhou Xiao, Huayue Yu, Xia Jin, Songqing Fan, Wei Liu

**Affiliations:** ^1^ Department of Pathology, Hunan Cancer Hospital, The Affiliated Cancer Hospital of The Xiangya School of Medicine, Central South University, Changsha, China; ^2^ Department of Pathology, The Second Xiangya Hospital, Central South University, Changsha, China

**Keywords:** immunotherapy, insulin-like growth factor, metabolism, immune checkpoint inhibitors, IL-6, EGFR, LIN28-let7 axis, HNSCC

## Abstract

The development of techniques and immunotherapies are widely applied in cancer treatment such as checkpoint inhibitors, adoptive cell therapy, and cancer vaccines apart from radiation therapy, surgery, and chemotherapy give enduring anti-tumor effects. Minority people utilize single-agent immunotherapy, and most people adopt multiple-agent immunotherapy. The difficulties are resolved by including the biomarkers to choose the non-responders’ and responders’ potentials. The possibility of the potential complications and side effects are examined to improve cancer therapy effects. The Head and Neck Squamous Cell Carcinoma (HNSCC) is analyzed with the help of programmed cell death ligand 1 (PD-L1) and Insulin-like growth factor (IGF). But how IGF and PD-L1 upregulation depends on IL-6, EGFR, and LIN28/Let7-related mechanisms are poorly understood. Briefly, IL-6 stimulates gene expressions of IGF-1/2, and IL-6 cross-activates IGF-1R signaling, NF-κB, and STAT3. NF-κB, up-regulating PD-L1 expressions. IL-6/JAK1 primes PD-L1 for STT3-mediated PD-L1 glycosylation, stabilizes PD-L1 and trafficks it to the cell surface. Moreover, ΔNp63 is predominantly overexpressed over TAp63 in HNSCC, elevates circulating IGF-1 levels by repressing IGFBP3, and activates insulin receptor substrate 1 (IRS1).TP63 and SOX2 form a complex with CCAT1 to promote EGFR expression. EGFR activation through EGF binding extends STAT3 activation, and EGFR and its downstream signaling prolong PD-L1 mRNA half-life. PLC-γ1 binding to a cytoplasmic motif of elevated PD-L1 improves EGF-induced activation of inositol 1,4,5-tri-phosphate (IP3), and diacylglycerol (DAG) subsequently elevates RAC1-GTP. RAC1-GTP was convincingly demonstrated to induce the autocrine production and action of IL-6/IL-6R, forming a feedback loop for IGF and PD-L1 upregulation. Furthermore, the LIN28-Let7 axis mediates the NF-κB-IL-6-STAT3 amplification loop, activated LIN28-Let7 axis up-regulates RAS, AKT, IL-6, IGF-1/2, IGF-1R, Myc, and PD-L1, plays pivotal roles in IGF-1R activation and Myc, NF-κB, STAT3 concomitant activation. Therefore, based on a detailed mechanisms review, our article firstly reveals that IL-6, EGFR, and LIN28/Let7-related mechanisms mediate PD-L1 and IGF upregulation in HNSCC, which comprehensively influences immunity, inflammation, metabolism, and metastasis in the tumor microenvironment, and might be fundamental for overcoming therapy resistance.

## 1 Introduction

Squamous cell carcinoma is the most common kind of head and neck tumor, and it may develop in several anatomical locations, including the pharynx, oral cavity, and larynx. Over 890,000 new cases and 450,000 deaths were attributed to HNSCC in 2018, making it the sixth most frequent cancer worldwide (1). Long-term smoking, heavy alcohol use, and infection of the human papillomavirus or the Epstein-Barr virus are the main risk factors and oncogenic drivers worldwide (1). There have been improvements in the treatment of HNSCC over the last several decades, but in general, most patients are still given a combination of surgery, radiation, and chemotherapy. About half of all cases are detected at an advanced stage because of the significant difficulties in early diagnosis. Despite developing EGFR-targeted treatment and PD-1/PD-L1 blockade immunotherapy, the 5-year survival rate has remained around 50% in advanced patients (1). To enhance patient care, new treatment methods must be developed. This cannot be done without understanding the particular, detailed processes and co-occurrence of the oncogenic changes of main pathways in the HNSCC microenvironment (2). As a kind of immunotherapy for cancer, targeted antibodies may interfere with cancer cell function and trigger the immune system to target and destroy cancer cells. Some immunotherapy medications, known as immune checkpoint inhibitors, prevent checkpoint proteins from engaging in protein-protein binding. Due to this, the off signal is not sent, and the T cells can effectively eliminate cancer cells. The CTLA-4 checkpoint protein is a target for one such medication. Toxic effects on the immune system or other systems as a consequence of immune system dysfunction are collectively referred to as immunotoxicity.

The Insulin-like development factors system consists of IGF-1 and -2, their receptors (IGF-1R and -2R), and a group of six IGF-binding proteins (IGFBP-1 to -6) (3: 4). Specifically, there is some cross-activation between insulin and IGF-1, IGF-2, and their receptors (5, 6) since IGF1R has a high degree of similarity with the insulin receptor. To interact with their substrates, the IRS and the Src homology 2 domain-containing transforming protein (SH2), IGF-1/2 receptor tyrosine kinase (RTK) activity may be activated by many different stimuli. Phosphorylation of this protein causes it to behave as a docking molecule, activate the RAS/RAF/MEK and PI3K/AKT/mTOR signaling pathways, stimulate cell proliferation, and suppress apoptosis, all of which contribute to carcinogenesis and resistance to numerous therapies (7, 8). PD-L1 overexpression was demonstrated to activate IGF-1R-mediated PI3K/Akt and RAF/MEK/ERK pathways and elevate STAT3, NF-κB, and c-Myc, which are reversible by lycopene in tongue squamous cell carcinoma (9). Moreover, PD-L1 upregulation elicits immune evasion in HNSCC, correlates with Let7 downregulation, PD-L1 expression in HNSCC is heavily glycosylated, Let-7a/let-7b miRNA was shown to promote PD-L1 degradation and inhibit PD-L1 glycosylation (10). IL-6 was found to regulate PD-L1 mutually, and its elevation correlated with disease failure and poor prognosis for HNSCC (11–13). We shall review the oncogenic IL-6/JAK/STAT3, EGFR/RAS/MEK/ERK, PTEN/PI3K/Akt pathways in HNSCC and LIN28/Let7 axis they regulate PD-L1 expression and degradation. Anti-tumor efficacy may be measured in several ways. Still, regardless of the host species, the ultimate objective of any therapy should be to reduce tumor burden, minimize tumor-associated morbidity, enhance the quality of life, and, if feasible, increase the life span. Direct destruction of cancer cells or encouragement of the immune system to fight against the tumor are two possible mechanisms behind its anti-tumor impact. Exposure to potentially toxic compounds, such as chemical warfare agents, may have detrimental consequences on immune function, known as immunotoxicity.

The antitumor immune response is remarkably clinically effective when the PD-L1. programmed cell death receptor-1 (PD-1) signaling axis is inhibited. Low response rates, however, only allow a small subset of patients to benefit from immunotherapy. In recent investigations, the significance of the transmembrane protein PD-L1 in exosomes has been investigated, and it has been discovered that exosomal PD-L1 functions as a mechanism of tumor immune escape and immunotherapy resistance. Exosomal PD-L1 transmits functional PD-L1 across the tumor microenvironment, reduces T cell effector function, and causes systemic immunosuppression (TME). Exosomal PD-L1 has been suggested as a biomarker to predict immunotherapy response and to evaluate treatment efficacy due to its substantial role in immune evasion (14).

**Figure 1:**
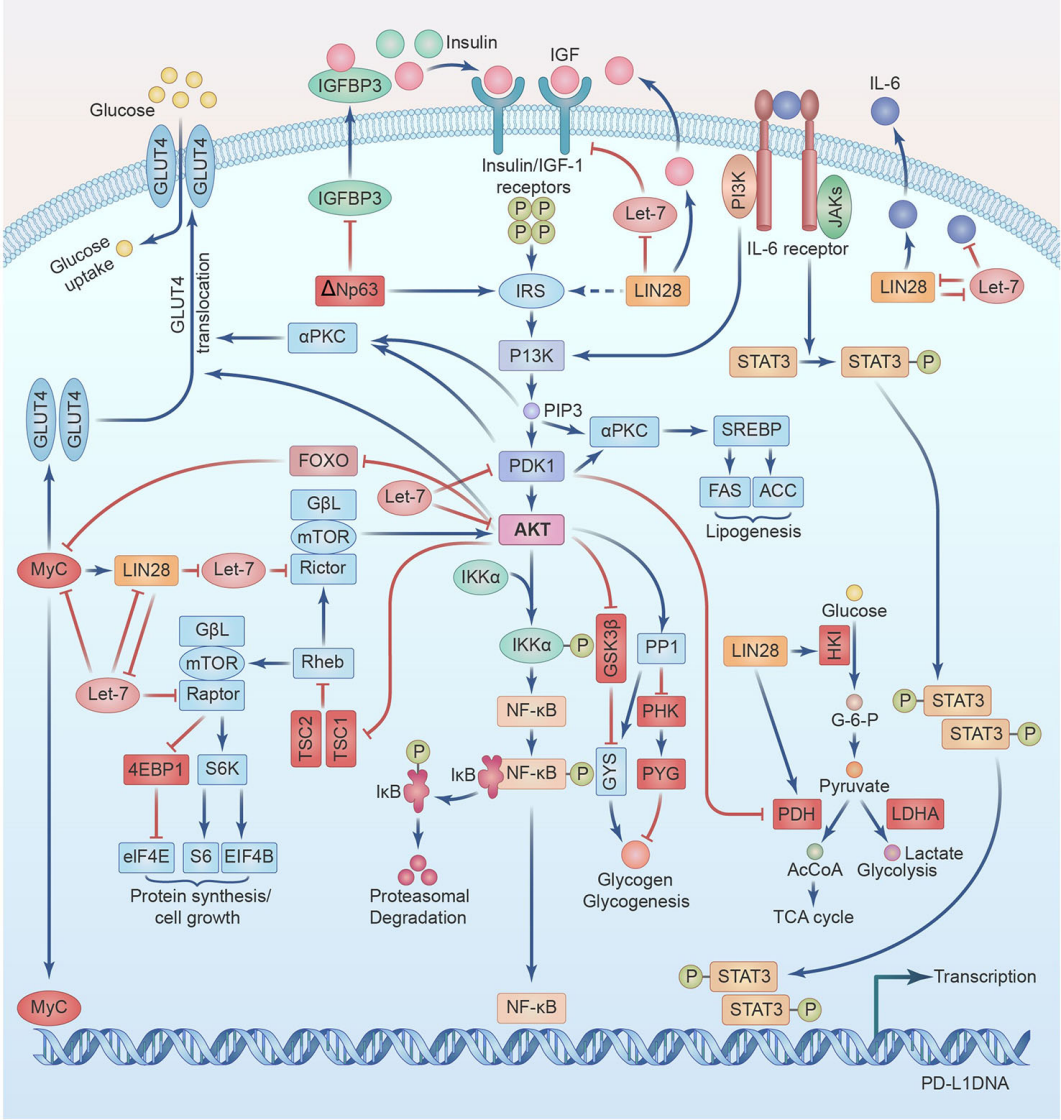
IGF signaling cross-talks with PD-L1 regulation through LIN28-Let7 axis and IL-6 amplification loop in Head and Neck Squamous Cell Cancer.

The development of several types of solid tumors, including breast cancer (BC) and head and neck squamous cell carcinoma(HNSCC), as well as a number of pathophysiological processes has been linked to the insulin-like growth factor-1 (IGF-1) over the past 20 years, according to numerous research. IGF-1 receptor (R) is overexpressed and hyperphosphorylated in a number of BC subtypes, according to preclinical and clinical studies. This pathway is a key player in the proliferation and spread of tumor cells, making it a crucial therapeutic target. Moreover, the IGF-1 axis has demonstrated a close relationship with the control of estrogen and endocrine therapy, suggesting a potential cure for anti-estrogen resistance. Many clinical trials are currently examining the function of IGF-1R inhibition in regulating resistance mechanisms to target therapies. IGF-1R may potentially interact with other essential therapeutic approaches, such as anti-HER2 medicines and mTOR inhibitors. With an eye on their potential future influence on clinical practice, our goal is to provide a summary of the most current and significant field of application for IGF-1 inhibitors as well as pertinent treatment approaches (1). We will review them in full detail below, emphasizing their connections with PD-L1 and providing possible explanations for PD-L1 overexpression-induced effects and IGF-1R activation (see **Figure 1**).

## 2 IL-6/JAK and STAT3

### 2.1 IL-6/JAK

IL-6 is a pleiotropic cytokine involved in many physiological processes and a prominent participant in chronic inflammatory disorders, autoimmune diseases, cytokine storms, and cancer (15). IL-6 was identified in 1986 and is situated on the short arm of chromosome 7 (16). Multiple cell types, including B- and T- lymphocytes, monocytes, fibrocytes, endothelial cells, keratinocytes, adipocytes, and cancer cells, can produce and secrete IL-6 (17). IL-6 is also involved in menstruation and spermatogenesis, bone metabolism through the stimulation of osteoclastogenesis and osteoclast activity (18), liver regeneration (19), and tissue-protective/reparative and inflammatory characteristics of vascular endothelial cells (20).

Many patients with hematopoietic malignancies or solid tumors have been shown to have elevated levels of IL-6 from tumor cells, stromal cells, and tumor-infiltrating immune cells, which has been linked to poor outcomes (13). Both membrane-bound IL-6R (the classical signalling) and soluble(s) IL-6R (the trans-signalling) are involved in the transmission of IL-6 signals. While IL-6 always works the same way, first binding to the receptor and subsequently to gp130 through cytokine binding domains, the diverse receptor types dictate distinct biological effects (17). Researchers believe only immune cells, liver cells, intestinal epithelial cells, and vascular endothelial cells express IL-6R and benefit from its anti-inflammatory effects (20). The cell surface IL-6R is cleaved into sIL-6R by the proteases disintegrin and ADAM17 and ADAM10(17).

Interestingly, ADAM17 or ADAM10 cleaves PD-L1 off the malignant cells and extracellular vesicle surface to form soluble but still active PD-L1, which impairs the destruction of tumor cells and promotes the death of CD8+ T cells (21). After sIL-6R forms a dimer complex with IL-6, the complex is transported throughout the body. The IL-6-SIL-6R complex forms a heterotrimer, which binds to gp130 to create a hexameric complex with another heterotrimer. The sIL-6R shedding by tumor-infiltrating T cells, neutrophils, and monocytes allows IL-6 to stimulate trans-signaling in tumor and stromal cells, resulting in oncogenic pro-inflammatory responses (17). Since gp130 is ubiquitous, IL-6 can be produced by tumor cells, infiltrating immune cells and stromal cells.

Full activation of JAKs occurs after IL-6-(s) IL-6R complex engagement of gp130, which disrupts JAK homology2 domain (JH2)-mediated regulation of JH1 domain activity by mutual transphosphorylation (17). Since the genes for JAKs, both upstream kinases that control PD-L1 expression, are located on the same chromosome (9p), mutation and amplification of the JAK family lead to increased PD-L1 transcription (22). PD-L1 is protected against GSK3-26S proteasome-driven degradation, improves its association with PD-1 on CD8+ T cells, and mediates EMT, as shown in another study (23). This is accomplished by priming PD-L1 for glycosylation of the catalytic subunit of oligosaccharide transferase STT3. Fully glycosylated PD-L1 has a half-life of 12 h (24), but nonglycosylated PD-L1 has a half-life of 4 h and is quickly proteolyzed. Multiple tyrosines remaining in the cytoplasmic region of gp130 are phosphorylated by active JAK enzymes, transforming them into docking sites that bring proteins into close contact with the enzymes. The proteins are phosphorylated by the active JAK enzymes, which then set off the JAK/STAT3, AKT/PI3K, and RAS/RAF/MEK/MAPK signaling pathway (16). These three signaling pathways, which may also be triggered by EGFR, IGF-1R, and HIF and contribute to PD-L1 regulation, are responsible for the hexameric complex’s ability to elicit anti-inflammatory or pro-inflammatory responses (25).

AP-1, C/EBP, and cAMP-responsive elements are only some transcription factors that bind to specific sites in the IL-6 gene (15). One of the main drivers of IL-6 expression and secretion is NF-κB, which is partially activated *via* the PI3K/Akt/mTOR pathway. IL-6 induces JAK activation, stimulating STAT3 dimerization *via* binding to sIL-6R, which subsequently binds to gp130. STAT3’s capacity to increase IL-6 gene expression creates an autocrine feed-forward loop. An amplification loop is formed when IL-6 in the tumor microenvironment activates STAT3 and NF-кB, which then increases the production of IL-6 (17). Perhaps this is the reason why IL-6 levels are so high in HNSCC. The direct effects on tumor cell survival, propagation, and incursion, increased IL-6 levels can also induce the appearance of other growth cytokines or factors significant for HNSCC progression and development, like IGF, GM-CSF, VEGF, monocyte chemoattractant protein (MCP)1, CCL3, CCL2, CCL5, IL-1β, and IL-8, which act in a paracrine or autocrine fashion on both immune and nonimmune cells within the tumor microenvironments (26). Elevated IL-6 levels drive the STAT3, MAPK, PI3K/Akt pathways activation, and NF-κB, which leads to diverse pathological chronic inflammatory diseases like cancer, multiple sclerosis, rheumatoid arthritis, diabetes, Castleman disease, inflammatory bowel disease, Crohn’s and Alzheimer’s disease; the clinical usage of IL-6 signaling inhibitors have proved helpful in the treatment of patients with these disorders (15, 17).

Head and neck cancer and pancreatic cancer cell lines rank the highest according to the cancer inflammation indices, based on IL-6 and PD-L1 genes commonly binding-factors NF-κB, STAT3, and AP-1 (27) and correlated IL-1/NF-κB, IL-6/STAT3, and TNF/AP-1 signaling cascade (28). Together, these constituents provide a much larger positive feedback loop that sustains the altered malignant state (28). An illuminating investigation on non-small-cell lung cancer found an IL-6/STAT3/IGF-1R autocrine loop, in which IL-6 stimulates the gene production of IL-6 itself, as well as IGF-1 and IGF-2, which cross-activate IGF-IR and STAT3, resulting in EMT and an increase in IGF-1R promoter activity (29). IL-6 was also confirmed to stimulate an autocrine or paracrine IGF-1/IGF-1R expression which, through STAT3, stimulated OCT4/NANOG expression in hepatitis B virus-infected hepatocellular carcinoma (30). These findings support that IL-6 mediates PD-L1 and IGF elevation.

### 2.2 STAT3

The high levels of STAT3 expression in the pericardium, leukocytes, peritoneum, mammary gland, digestive tract, kidney, bladder, lung, bone marrow, and peripheral nervous system all point to critical roles for STAT3 in these organs’ physiological processes (31). The activation of STAT3 is tightly controlled in healthy cells. STAT3 phosphorylation induced by exposure to cytokines peaks within 15–60 minutes and decreases over several hours. Phosphorylated STAT3 returns to the cytoplasm through nuclear pores after being dephosphorylated by nuclear protein-tyrosine phosphatases (PTPs) such as TC-PTP or TC45 (32). Moreover, several other negative protein regulators exist, including suppressor of cytokine signaling proteins (SOCS1-7); the PIAS1-4; several PTPs, including SHP-1, SHP-2, PTPRD, PTPRT, DUSP22, and CD45; and the ubiquitination-dependent proteasomal degradation systems (33). SOCS family negatively regulates the STAT/JAK pathway in terms of preventing the tyrosine kinase receptor and straight binding to JAK or simultaneously binding JAK with gp130 to inhibit JAK kinase activity, (3) targeting SOCS-bound JAK or STAT for proteasomal degradation *via* polyubiquitination by forming a complex with the elongation protein B/C-cullin5 complex. For example, SOCS3 silencing by CpG islands hypermethylation of the promoter in human lung cancer results in STAT3 hyperactivation and tumor cell proliferation (34). PIAS does not interact with STAT monomers but only inhibits formed STAT dimers after JAK phosphorylation. PIAS mainly regulates transcription by blocking STAT DNA-binding activity, promoting transcript factor sumoylation, and recruiting transcriptional co-repressors such as histone deacetylase (35). PTPs can dephosphorylate STAT and therefore inhibit JAK/STAT signal transduction. SHP1 and SHP2 are examples of tyrosine phosphatases that include the SH2 domain. While SHP2 is expressed in all cell types, SHP1 is mostly found in hematopoietic, smooth muscle, and epithelial cells. Dephosphorylation of active JAKs or STATs occurs when their SH2 domains bind to phospho-tyrosine residues (36). Selected PTPRT mutations correlate with up-regulation of phospho-STAT3 Y705 expression in HNSCC specimens and cell lines, whereas overexpression of wild type reduced STAT3 phosphorylation. Loss of expression of PTPRT and PTPRD occurs due to mutations in these genes in 3.7% and 5.6% of neck and head cancers, respectively, and promoter methylation in 60% of HNSCC cases (17). Cell proliferation is stimulated, and the expression of the inhibitory target phospho-STAT3 Y705 is elevated in response to PTPRD mutations that abolish its action (36). Primary melanoma cells lacking endogenous PTPRD show markedly reduced cell growth and death when treated with exogenous PTPRD (37).

STATs are activated *via* the tyrosine phosphorylation cascade after binding and stimulation by ligands, including members of the IL-6 cytokine receptor family, such as IL-6, CNTF, LIF, and OSM (15); interferon (IFN-α/β/γ); RTKs, such as EGFRs, VEGFRs, IGFRs, the insulin receptor, c-Kit, and c-Met (38); and nonreceptor tyrosine kinases (NRTKs), such as JAKs and Src family kinases (SFKs). GPCRs, like the sphingosine 1 phosphate receptor 1/2 (S1PR1/2) and angiotensin II (Ang II), and some TLRs, like TLR 2, 3, 4, 7, and 9, can also mediate STAT3 activation (39). STAT3 mutations are rare only in patients with hematological malignancies (17). The hyperactivation of growth factor receptors/NRTKs, including Src/JAKs; the overexpression of stimulatory ligands, such as EGF/IL-6; or the loss of function of negative regulators, such as SOCS/PIAS/PTPs, lead to aberrantly elevated STAT3 activity in over 70% of human cancers (39). STAT3 translocates into the nucleus *via* an importin/importin 1-dependent process after being phosphorylated at Tyr705 (40). This occurs either through SH2-domain-mediated head-to-tail dimerization or the formation of a dimer with STAT1. The STAT3 dimer binds gamma-activated sites in the promoters of a wide variety of target genes in the nucleus of various cancer cells, including head and neck cancer cells and multiple myeloma (41), influencing the transcription of genes important for cellular proliferation (MYC and cyclin D1), survival (survivin and BCL-xL), angiogenesis (such as VEGF), and maintenance of the immunosuppressive micro-environment. In addition, tumor microenvironmental STAT3 hyperactivation promotes metastasis and cancer progression by elevating the appearance of EMT-related transcription elements such as Twist, Snail, and ZEB1 (42).

STAT3 also underlies resistance to multiple therapies, such as conventional chemotherapy, radiation therapy, and EGFR-targeted therapies; therefore, combination therapy with STAT3 inhibitors may be of clinical benefit (43). Recent elegant research exposed that bone marrow-derived mesenchymal stem cells (BM-MSCs) elicited early-response CD133+/CD83+ lung cancer stem cell (CSC) dissemination and, through LIF/LIFR/pS727-STAT3 signaling, induced mesenchymal-epithelial transition (MET) to establish a macroscopic premetastatic niche. Then, BM-MSCs aided primary cancer cell EMT through IL-6/IL-6R/pS705-STAT3 signaling, leading these cells to acquire CD151+/CD38+ CSC properties and to be attracted to the premetastatic niche. Therefore, the IL-6 cytokine family receptor/JAK/STAT3 axis targeting might also block two-step cancer metastasis and reverse the dissemination of metastasis-related CSCs (44). Microsatellite instability/defective mismatch repair (MSI/dMMR), programmed death ligand 1 (PD-L1), and tumor mutational load are some of the most extensively studied prognostic biomarkers for immunotherapy (TMB). Many preclinical investigations and clinical trials have focused on individuals who have developed a primary or acquired resistance to ICI because of the difficulty of treating this condition. Immune-related adverse events (irAEs) are a distinct spectrum of ICI side effects that mimic autoimmune reactions. The skin, digestive tract, lungs, endocrine, musculoskeletal, and other systems are among the most prevalent sites where irAEs manifest themselves, although they may affect any organ in the body. The current body of data shows that the extreme reserve of TCR inhibitory phosphatases linked with CPI receptors, which are responsible for maintaining peripheral immunological tolerance towards autoantigens, may be the primary cause of irAEs in patients treated with CPI. Active and passive approaches are the two primary categories of cancer immunotherapy to enhance anti-tumor efficacy while abrogating toxicities. Cancer elimination is enhanced by interventions that boost the patient’s immune system, such as vaccination or adjuvant therapy.

## 3 EGFR/PD-L1

EGFR is a 170 kDa transmembrane glycoprotein and RTK that is an associate of the ErbB/HER family with ErbB3 (HER3), ErbB2 (HER2/neu), and ErbB4 (HER4). Except for HER2/neu, ligands have been identified for all HER family members. Seven ligands for EGFR are known, including the four high-affinity ligands EGF, heparin-binding EGF, TGF-α, and amphiregulin, and the three low-affinity ligands epiregulin, betacellulin, and epigen. When a ligand hits EGFR, it stimulates homo- or heterodimerization of EGFR with additional HER2, HER3 receptors or other RTKs, such as IGF-1R or MET (45). Dimerization of the extracellular region of the receptor induces intracellular conformation changes, which releases intramolecular cis-autoinhibition, resulting in the transphosphorylation of key tyrosine residues, which allows the kinase to assume an active conformation (46). EGFR activates four downstream signaling cascades: the MAPK, PI3K/AKT/mTOR, JAK/STAT, and PLCγ/PKC pathways. Dysregulation of these pathways is commonly observed in the occurrence and metastasis of many cancer types, especially HNSCC (47). A study in SkHep-1 cells revealed that EGF, TGF-α, HB-EGF, and beta-cellulin stimulation, but not amphiregulin or epiregulin, caused dose-dependent EGFR accumulation in the nucleus. Stimulation with the former four ligands also increased the phosphorylation of EGFR tyrosine residues and wound closure rate more than stimulation with the latter two ligands. However, a single ligand stimulation failed to promote proliferation (48). An informative study revealed that unlike new PD-L1 transcription mediated by interferon regulatory factor 1 interaction with the PD-L1 promoter succeeding IFN-γ activates JAK/STAT axis, the binding EGF to EGFR elevates PD-L1 transcript levels by prolonging the mRNA half-life. PD-L1 overexpression in HeLa cells promoted migration and invasion; doubled the thymidine incorporation into DNA; and facilitated EMT by increasing SNAIL, SLUG, and ZEB1 levels, and H1975 cells overexpressing PD-L1 displayed more robust tumorigenesis in athymic mice. PD-L1 deletion or PD-L1 antibodies abrogated EGF and TGF-β -inducible migration, tumorigenesis, and metastasis (49). Most interestingly, the authors created an EGF gradient and measured directionality that connects the linear distance between the starting point and endpoint (D) to the total distance (T) traveled. PD-L1 deficiency or application of the EGFR kinase inhibitors significantly reduced persistence. As actin filaments and Rho family GTPases control the switch from random to persistent migration, PD-L1 ablation caused a reduction in RAC1-GTP and CDC42-GTP levels but elevated RHOA-GTP levels with visually severe loss of actin cables (49). Most importantly, RAC1-GTP was convincingly demonstrated to induce the autocrine production and action of IL-6/IL-6R, leading to STAT3 activation, which might induce PD-L1 expression and EMT, linking PD-L1 signaling with regulation (50–52). The cytoplasmic tail of PD-L1 was cloned into a yeast 2-hybrid bait construct, and >50 million connections have been examined to discover direct PD-L1 binders. The breakthrough occurred when filamin A and PLC-γ1 were identified as the two PD-L1 interaction partners with the maximum confidence scores (49). Each monomer of the F-actin crosslinking protein Filamin A consists of an actin-binding domain and a rod segment. Filamin A dimers crosslink actin filaments to create networks orthogonal to parallel bundles, while the two hinges in the rod section allow for the inherent flexibility of actin networks. Briefly, the distinctive structure of filamin A and its specific interaction with F-actin confer dynamic mechanical features on the cytoskeleton (51). The binding of PLC-γ1 to a conserved cytoplasmic domain of PD-L1 promotes EGF-induced activation, resulting in the production of DAG and IP3. The former concentration-dependently activates PKC-, while the latter increases cytosolic calcium ion levels and regulates actin-myosin contractions. Notably, PLC-γ1 was not activated when EGFR was inactive, or PD-L1 was scarce; this process is known as the EGFR and PD-L1 two-hit model of PLC- activation. The attachment of PLC-γ1 to the cytoplasmic region of PD-L1 has also been associated with protection against IFN-γ cytotoxicity (49).

A previous report revealed that the genomic binding sites of master transcription factors TP63 and SOX2 in SCCs overlap. An epigenomic profiling of various SCCs, including HNSCC, demonstrated that SOX2 and TP63 cooperatively bind and activate the super-enhancers and promoter of the long non-coding RNA (lncRNA) CCAT1 to regulate its expression. ChIP analysis showed that oncogenic CCAT1 forms a multifaceted with SOX2 and TP63, which binds the super-enhancers of EGFR to regulate EGFR expression, thereby activating both PD-L1-related MEK/ERK1/2 and PI3K/AKT signaling pathways (53). In light of TP63, ΔNp63, which is predominantly overexpressed over TAp63 in HNSCC (27), has been shown to elevate circulating IGF-1 levels by repressing IGFBP3 and increasing intracellular MEK/ERK1/2 and PI3K/AKT/mTOR signaling by activating IRS1 expression, ultimately promoting the growth and survival of SCC cells (54). SOX2 was found to bind a proximal site in the Lin28 promoter region and positively regulate Lin28 expression in neural precursor cells (55). Lin28 elevates IGF-2 and IL-6, activates IGF-1R and Myc, and enables the activation of NF-kB, STAT3, and c-Myc to elevate PD-L1 expression (56). EGFR mutations were demonstrated to upregulation PD-L1 expression *via* the IL-6/STAT3/JAK pathway in non-small cell lung cancer, mediating tyrosine kinase inhibitors (TKIs) resistance (57). PD-L1 recruits phospholipase C-γ1 to its cytoplasmic motif, which enhances phospholipase C-γ1 activation by EGFR and tumorigenicity of EGFR-mutant lung tumors (49). Matrine and Polyphyllin I overcomes EMT-associated resistance to TKI *via* IL-6/STAT3 pathway inhibition in lung cancer (58, 59). Non-coding RNA miR-146b concurrently blocked both the IL-6-STAT3 and EGFR pathways *in vitro* and might be of clinical benefit in ovarian cancer (60). Reports that lycopene reverses PD-L1 expression and signaling in HNSCC and that tomato lectin specifically blocks EGFR signaling-mediated poly-LacNAc glycosylation necessary for the PD-L1/PD-1 interaction in triple-negative breast cancer (TNBC) might provide some hope (58; 61). These results identified an SCC-specific DNA/RNA/protein multifaceted that stimulates IGF signaling and upregulates PD-L1, progressing our understanding of metastasis and immunity in cancer biology.

EGFR is a crucial switch in the activation of STAT3 by IL-6. SOCS3 may directly block the kinase activity of receptor-associated JAK proteins, preventing subsequent activation of STAT3. EGFR activation can inhibit SOCS3 and nullify the negative control of JAK kinase activity (62). IL-6 stimulates the shedding of EGFR ligands through ADAM10 and ADAM17, so it transactivates EGFR in normal prostate epithelial cells and promotes cell proliferation (63). IGFBP2 is a secreted protein that controls the bioavailability and localization of IGF-1 and IGF-2 by binding to them. IGFBP2 was a possible biomarker for PI3K/Akt pathway activation and PTEN status, and it was demonstrated to upregulate PD-L1 expression in glioma, prostate, breast, colorectal, and HNSCC (64). IGFBP2 and EGFR nuclear colocalization were reported in glioblastoma and astrocytoma cells. Additional research demonstrated that IGFBP2 enhances the EGFR nuclear accumulation and the activation of the EGFR/STAT3/PD-L1 signaling pathway in melanoma cells (65). IGFBP2 nuclear translocation in cancer cells is based on a typical importin-α-dependent mechanism mediated by a nuclear localization signal (179PKKLRPP185) in the linker domain of IGFBP2 (64).

**Figure 2:**
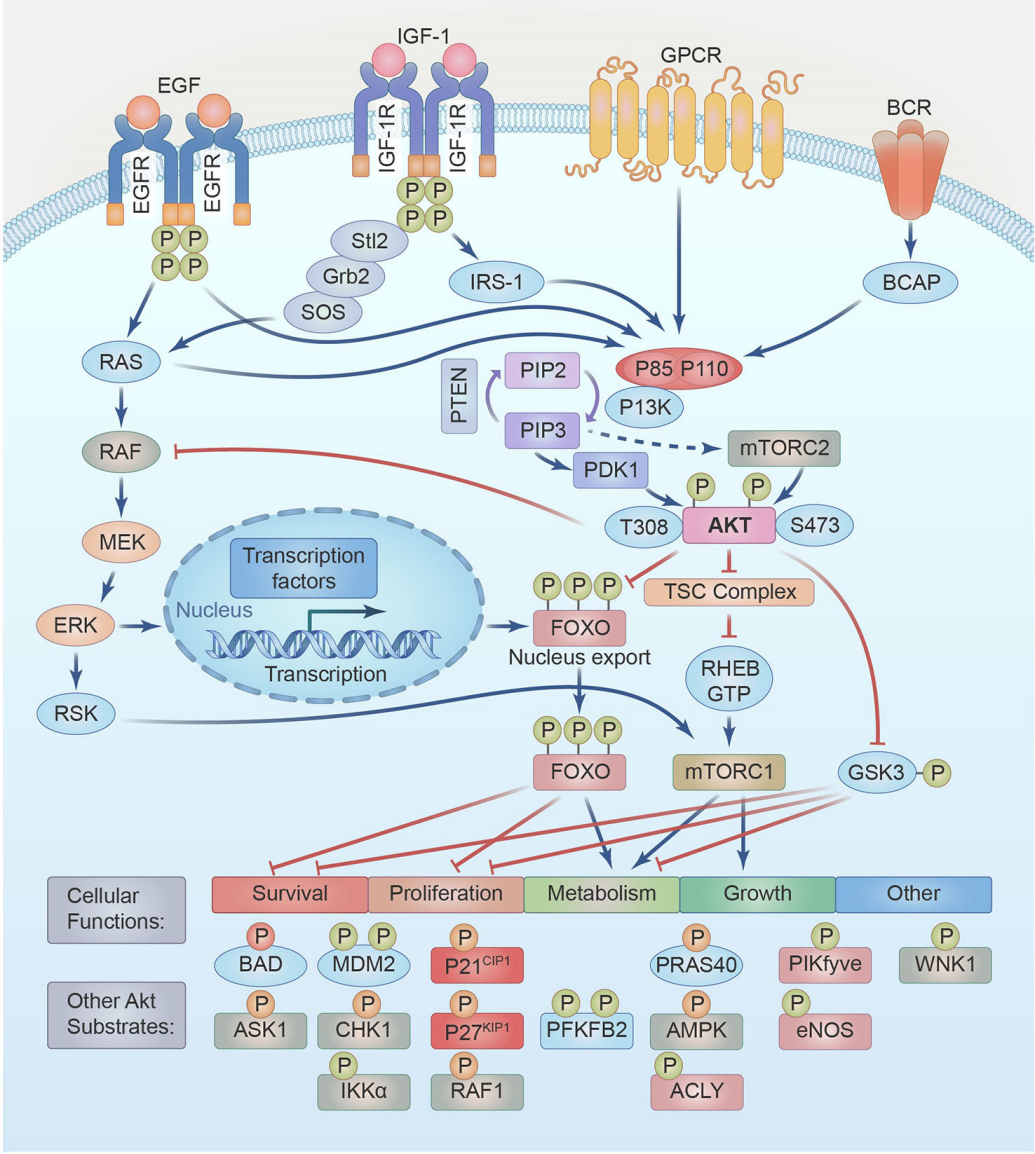
IGF/IGFR,EGF/EGFR,GPCR,BCR signal through PI3K-AKT-mTOR and RAS-RAF-MEK-ERK pathways. A partial list of their functions and substrates are shown.

The RAS/RAF/MEK/ERK signal transduction pathway activates numerous critical cellular mediators, leading to proliferation, cell growth, differentiation, invasion, migration, and survival (64). HRAS, KRAS, and NRAS are the three members of the Ras gene family. Ras is a GTPase localized on the intracellular side of the plasma membrane that RTKs, including EGFR, activate. Guanine nucleotide exchange factors (GEFs), which permit GTP loading, influence the transition of Ras from an inactive to an active state. GTP hydrolysis mediates the reverse switch, which is mediated by GTPase-activating proteins (GAPs) (66). Once Ras is active, it stimulates the RAF (RAF1) and PI3K pathways (45). RAF has three variants: ARAF, BRAF, and CRAF (RAF1). GTP-bound RAS interacts with and recruits RAF, accumulating RAF at the plasma membrane and subsequent dimerization, activating RAF kinases. Although all three isoforms activate MEK, data suggest that oncogenic RAS may activate them differently. Activated RAF phosphorylates MEK1 and MEK2 kinases, activating the MAP kinases ERK1 and ERK2. These MAP kinases may either phosphorylate cytoplasmic substrates or translocate into the nucleus to target genes that govern cell growth, proliferation, and survival (66). Mutated RAS phosphorylation increases hyperactive MEK signaling and inhibits tristetraprolin, which usually prohibits PD-L1 and LIN28 mRNA by binding AU-rich regions, thereby extending their half-lives (67) (see **Figure 2**).

EGFR is overexpressed in 90% of HNSCC cases with pathway genetic alteration frequency at 42% correlates with poor prognosis and treatment outcomes (2). Malignant tumors are often addicted to EGFR signaling for sustained survival and proliferation, even after acquiring resistance to multiple therapies (68). Strategies targeting EGFR for the treatment of HNSCC have been widely evaluated. Their mechanisms are oral TKIs bind the intracellular tyrosine kinase domain of EGFR and prevent downstream signaling, intravenously administered antibodies bind the extracellular domain of EGFR to promote its internalization, also facilitate ADCC, which is mediated by NK cells, and antigen-presenting cells give rise to innate and adaptive antitumor immune responses *via* EGFR-specific T cells (68).

## 4 PTEN/PI3K/AKT/mTOR

6% of HNSCC tumors have reduced expression of PTEN or the loss of its function due to genetic and epigenetic alterations (2). PTEN was identified as a tumor suppressor for the first time in 1997, and its expression is usually diminished due to homozygous deletion in advanced cancers (69). Subsequent research identified PTEN as a strong adverse regulator of significant cell growth and survival signaling pathways, notably the PI3K/AKT/mTOR signaling pathways (70). Recent investigations have shown that PTEN also works as a key molecular switch that controls the reprogramming of cancer cell metabolism and autophagy (71). PTEN phosphatase decreases the activity of PI3K, hence impeding insulin/IGF-1 pathway signaling. Loss of PTEN function consistently underlying the pathogenesis of HNSCC by activating the PI3K/Akt/mTOR pathway, which increases PD-L1 protein translation ratios, as observed for glioma (72).

PTEN is a dual lipid and protein phosphatase, and its biological effects are driven by its capacity to dephosphorylate PIP3. In contrast, PTEN’s potential substrates include focal adhesion kinase (FAK), IRS1, c-SRC, and PTEN itself (73). When the p85α regulatory subunit of PI3K loses affinity for the p110α catalytic subunit, it’s binding to PTEN enhances its steadiness and phosphatase action (74). PI3K effector substrates, particularly AKT, become spontaneously activated without external oncogenic activation when PTEN is mutated or otherwise inactive (75).

PTEN regulates metabolic processes, including gluconeogenesis, glycolysis, glycogen synthesis, mitochondrial metabolism, and lipid metabolism (76). Cancer cells with inadequate PTEN expression manifest a glycolytic phenotype, termed the Warburg effect (77), by which metabolism is reprogrammed to match cell proliferation. In contrast, PTEN accumulation represses glycolysis and enhances oxidative phosphorylation by inhibiting the PI3K/Akt pathway (78, 79). The PTEN/PI3K/AKT axis regulates *de novo* lipogenesis by controlling sterol response element-binding protein (SREBP) expression and inhibiting the forkhead transcription factor FOXO1, which negatively regulates SREBP transcription and vice versa (80, 81). Notably, SREBP was also confirmed to be involved in PD-L1-mediated EMT, and overexpression of PD-L1 enhanced SREBP-1c promoter activity, which might result from the inhibition of FOXO1 (81–83).

PTEN also has important tumor-suppressor functions in the nucleus, mainly *via* its participation in various processes, including genome maintenance, DNA replication, DNA repair, cell cycle control, and gene expression (78). In prostate cancer, it was discovered that the X-linked tumor suppressor USP11 regulates protein stability and PTEN polyubiquitination in both the cytoplasm and nucleus (84). Mechanistically, PTEN inhibits PI3K/AKT, leading to FOXO1 dephosphorylation and nuclear translocation, activating the binding of FOXO1 to the USP11 promoter, which increases USP11 protein and mRNA levels to increase PTEN stability. This mechanism is exploited by mouse embryonic fibroblasts cultured at a high density to upregulate USP11 expression to increase PTEN protein levels for contact inhibition without mRNA changes. USP11 downregulation in cancer patients correlates with FOXO nuclear localization and PTEN expression. The PTEN-PI3K-FOXO-USP11 feed-forward loop improves the tumor-suppressive activity and stability of PTEN (84).

Overexpression of interleukin-8 (IL-8) correlates with poor outcomes in various cancers, including HNSCC, by activating the STAT3 pathway, which a CXCR1/2 inhibitor can block. More importantly, the binding of IL-8 to CXCR1/2 facilitates the phosphorylation and inactivation of PTEN, which functions as an important switch in the IL-8/STAT3 pathway, elevating snail expression and the autocrine/paracrine activity of IL-8 to form a novel positive feedback loop that might drive malignant progression (85). PTEN is also transcriptionally repressed by two PD-L1-related zinc finger-like transcription factors, SNAIL and SLUG, which compete with p53 for PTEN promoter binding (86). PTEN is regulated by non-coding RNAs and is important in adaptive and innate immunity; this is beyond the scope of this review and has been well-reviewed in the past (87, 88).

The PI3K/AKT/mTOR pathway is active in over 80% of HNSCC tumors because of EGFR activation (47%), PIK3CA mutations or amplifications (25%), PIK3R1 overexpression (2%) or PTEN mutation (6%) (2, 89). In HNSCC with wild-type PIKCA expression, prolonged tyrosine phosphorylation of PI3K and HER3 recruitment resulted in abnormal PI3K/AKT/mTOR signaling and immune suppression, as shown by an independent kinome-wide siRNA screen. HER3 antibody inhibition decreases PI3K/AKT/mTOR signaling, slows tumor development, and reverses the immunosuppressive tumor microenvironment (90). Activating the PI3K axis in HNSCC cells induces excessive proliferation, enhanced cancer cell survival, and resistance to several anticancer treatments (91). Recent studies have revealed that oncogenes may be activated by class I PI3K, AKT, and mTOR, contributing to the inhibition of autophagy and the promotion of cancer (92). PI3K axis activation is responsible for the immortal nature of HNSCC and might be conveniently exploited as an “Achilles heel” to improve therapeutic responses (93). In addition to EGFR, IGF1-R, HER2/neu, HER3, G protein-coupled receptors (GPCRs), and Ras GTPase, other RTKs can activate the PI3K/Akt signaling axis (91, 92). Different ligands, such as cytokines, homologous growth factors, and hormones, bind to the 58 RTKs from 20 different classes discovered in cells. Phosphorylation of RTK YXXM motifs upon ligand binding initiates signaling either directly through binding a regulatory component of PI3K (p85) or indirectly through adaptor proteins like IRS-1 (91). This interaction abrogates p85’s inhibition of p110, allowing PI3K to be activated. PIP2 is phosphorylated into PIP3 when PI3K is bound to the plasma membrane and activated (94). GPCRs are a huge family of cell surface receptors. Approximately 800 GPCRs and 35 G protein subunits transmit different signaling cascades and play crucial roles in several cellular and physiological functions (95). GPCRs may either straight activate PI3K by enhancing the kinase activity of p110β or circuitously by phosphorylating RasGEF and RasGRP4. The Gβγ subunits of GPCR bind with great affinity to a specific area in the C2-helical domain linker of p110β (96). RAS is another PI3K/Akt axis upstream activator (45). RAC1 and CDC42 binds directly to the N-terminal RAS-binding domain (RBD) of p110β and cooperates with RTKs to exert lipid kinase activity. After RTK activation, PIP3 propagates the PI3K/Akt signaling axes, activating the Rho complex to activate the PI3K enzyme through Ras-dependent p110β activation (97).

Once activated by the PIP3 binding to the PH domain, which modifies its configuration, AKT is recruited to the plasma membrane, where its phosphorylation is done at Thr308 by phosphoinositide-dependent protein kinase 1 (PDK1) (98). PIP3 also activates the mTORC2 complex by interacting with the PH domain within the SIN1 element and activated mTORC2 phosphorylates AKT at Ser 473 in the C-terminal hydrophobic motif, which is vital for high activation (99). IRS-1 and PI3K are recruited and activated by IGF-1-IGF-1R interaction. Activated PI3K transforms PIP2 to PIP3 as described above to activate AKT, and the dephosphorylation of AKT at Thr308 and Ser473 by protein phosphatase 2A (PP2A) modulates the activation state of AKT and its downstream effects (73). Activated AKT regulates multiple cellular processes by phosphorylating various targets, including mTORC1, TSC2, GSK3, FOXO, RAF1, IKKα, MDM2, CHK1, ASK1, p27, p21, BAD, PRAS40, eNOS, AMPK, and WNK1. These downstream effectors control important cellular processes, such as proliferation, transcription, metabolism, protein synthesis, cell survival, apoptosis, autophagy, angiogenesis, and EMT (see **Figure 2**). Phosphorylated AKT can activate mTORC1 either by direct phosphorylation at Ser2448 or by indirect phosphorylation and inhibition of TSC1/2 at Ser939 and Thr1462 (100), increasing protein, lipid, and nucleotide synthesis while downregulating autophagy, which supports cell survival, growth, and proliferation (91). Over C-terminal domains and GTPase-activating protein domains, TSC1 and TSC2 inhibit mTORC1 activity by converting Ras-related Rheb-GTP, strong mTORC1 activators, to Rheb-GDP. Inactivation of TSC2 maintains Rheb in its GTP-bound state, thereby augmenting mTORC1 activation (101). Activated Akt also inhibits the activity of both GSK-3α and GSK-3β by phosphorylating GSK-3α at Ser21 or GSK-3β at Ser9 in their N-terminal regulatory domains, which leads to different changes in transcription factors and upregulation of the anti-apoptotic BCL-2 family member MCL-1, transcription factors c-Myc, SREBP1c, β-catenin, HIF1α, glycogen synthase, and glucose transporter protein, etc. (100). Without inhibition by active AKT or p-EGFR, GSK-3β-mediated phosphorylation PD-L1 at T180 and S184 induces its relationship with the E3 ligase beta-transducin repeats-containing protein (b-TrCP), leading to PD-L1 degradation in the cytoplasm (102).

Other potent substrates of AKT are the Forkhead Box O transcription elements (FoxO1, 3, 4, and 6), which are generally considered to be tumor suppressors with established functions in DNA damage repair, the scavenging of reactive oxygen species, cell cycle arrest, apoptosis, metabolism, cell migration and angiogenesis (103). High nutrient availability or oncogenic activation of PI3K promotes Akt-mediated FOXO1 (Ser319, Thr24, and Ser256), FOXO3 (Ser315, Thr32, and Ser253), FOXO4 (Ser258, Thr28, and Ser193) and FOXO6 (Ser184 and Thr26) phosphorylation. This phosphorylation facilitates 14-3-3 chaperone protein binding, promoting FOXO nuclear export to the cytoplasm and preventing FOXO re-entry, blocking FOXO transcriptional activity. FOXOs accumulated in the cytosol can be polyubiquitinated and degraded *via* ubiquitin–proteasome pathway (103). Conversely, dephosphorylation mediated by PP2A rescues active AKT-phosphorylated FOXO1 and FOXO3 from nuclear exclusion and degradation (104). Extreme oxidative stress activates upstream c-Jun N-terminal kinase (JNK) and mammalian sterile 20-like kinase (MST1), JNK-mediated phosphorylation of FOXO4 (Thr447, Thr451) and FOXO3 (Ser574) and MST1-mediated phosphorylation of FOXO3 (Ser207) and FOXO1 (Ser212); promotes the capacity of FOXO proteins to control the expressions of target genes convoluted in survival (BIM and PUMA), cell growths (Sestrin3, MAP1LCB, and BNIP3), proliferation (p21 and p27), and metabolism (G6PC and PEPCK) is modulated (105,91) (see **Figure 2**).

Some studies have indicated that AKT signaling can phosphorylate the STAT3 protein, although it is sometimes unclear whether AKT phosphorylates STAT3 directly or indirectly through activating PKM2 or mTOR (105). Interestingly, IGF-1R signaling can activate STAT3 through interferon-induced transmembrane protein 2 (IFITM2) in gastric cancer (106), through the Stat3-Midkine-Stat3 loop in hepatocellular carcinoma (107), through the NF-κB-IL-6-STAT3 loop in non-small cell lung cancer (29), through IGFBP2-MDA-9 in prostate cancer (108), and RACK1 in ovarian cancer (109), suggesting the general need for an adaptor to mediate the activation of STAT3 by IGF-1R signaling. Determining whether this is the case in HNSCC needs careful investigation. Still, regardless, cytokine-mediated activation of STAT3 induces the expression of IGF-1R, so activation of the JAK/STAT3 pathway can cause insulin/IGF-1 signaling sensitization.

## 5 STAT3/NF-kB/c-Myc and PD-L1

Head and neck and pancreatic cancer cell lines rank the highest according to the cancer inflammation indices, which are based on IL-6 and PD-L1 genes commonly binding-factors NF-κB, STAT3, and AP-1 and their correlated IL-1/NF-κB, IL-6/STAT3, and TNF/AP-1 signaling cascades (28). The coactivation of NF-κB, STAT3, and AP-1 was confirmed in an updated TCGA report of HNSCC (27). Importantly, apart from mediating STAT3 activation *via* autocrine production and action of IL-6/IL-6R, activated Rac1 was reported to promote the deprivation of IκBα and the nuclear translocation of STAT3-NFκB multiplexes in starved cancer cells (110). Interestingly, cigarette smoke or cigarette smoke extracts (CSE) exposure was found to dose- and time-dependently elevate RAC1 expression and activity in mice or human bronchial epithelial cells under IL-8 and IL-6 expressions. CSE dose- and time-dependently persuaded the phosphorylation of STAT3, Erk1/2, and ERK1/2 or STAT3 inhibitor markedly reduced level of IL-8 and IL-6 after CSE exposure, inhibition of activity or expression of Rac1 blocked cigarette smoke or CSE-induced Erk1/2 and STAT3 phosphorylation *in vivo* or *in vitro* (111). Notably, oscillatory (1Hz) shear stress (10 dynes/cm2) activated RAC1 and CDC42 to stimulate the translocation of β-catenin to the nucleus and increase TCF/LEF activity in osteoblasts (112). STAT3 directly binds the IL-6 promoter to persuade IL-6 expressions. HPV was inveterate to drive the Rac1-NFκB-IL-6-STAT3 axis in cervical tumor to elevate IL-6 and STAT3, which correlates with cervical disease progression (113). NF-κB elevates LIN28 levels, inhibits Let-7 expression, and increases its target gene IL-6 expression, which activates NF-κB and STAT3, thereby forming positive feedback loops (56). This loop is reinforced by STAT3 activation, miR-181b, and miR-21, which are detected in the plasma of HNSCC patients (114) and can be up-regulated by STAT3 through direct binding to the promoter regions. miR-181b directly inhibits cylindromatosis (CYLD), a tumor suppressor and negative modulator of NF-κB, while miR-21 directly inhibits PTEN to activate PI3K-AKT- IKK-IκBs pathway. Both lead to NF-κB activation and act as a part of the epigenetic switch through the LIN28-Let-7 axis linking inflammation to cancer (56). PDLIM2, which is downregulated in HNSCC by systematic analysis of TACGA data, is a gene essential for NF-κB and STAT3 deprivation; normally, it improves the expression of proteins included in antigen presentation and endorses T-cell activation while inhibiting multidrug resistance genes, thereby renders mutated cells susceptible to immune observation and cytotoxicity (115). miR-221 subsidizes the constitutive activation of NF-κB and STAT3 by directly targeting and inhibiting PDLIM2 expression (116). Taken together, NF-κB and STAT3 co-activation occur in HNSCC through common induction by RAC1 and IL-6, common inhibition of degradation by PDLIM2, STAT3 indirectly reinforces NF-κB by inhibiting its negative modulator, and finally, co-binding to their common target genes.

**Figure 3:**
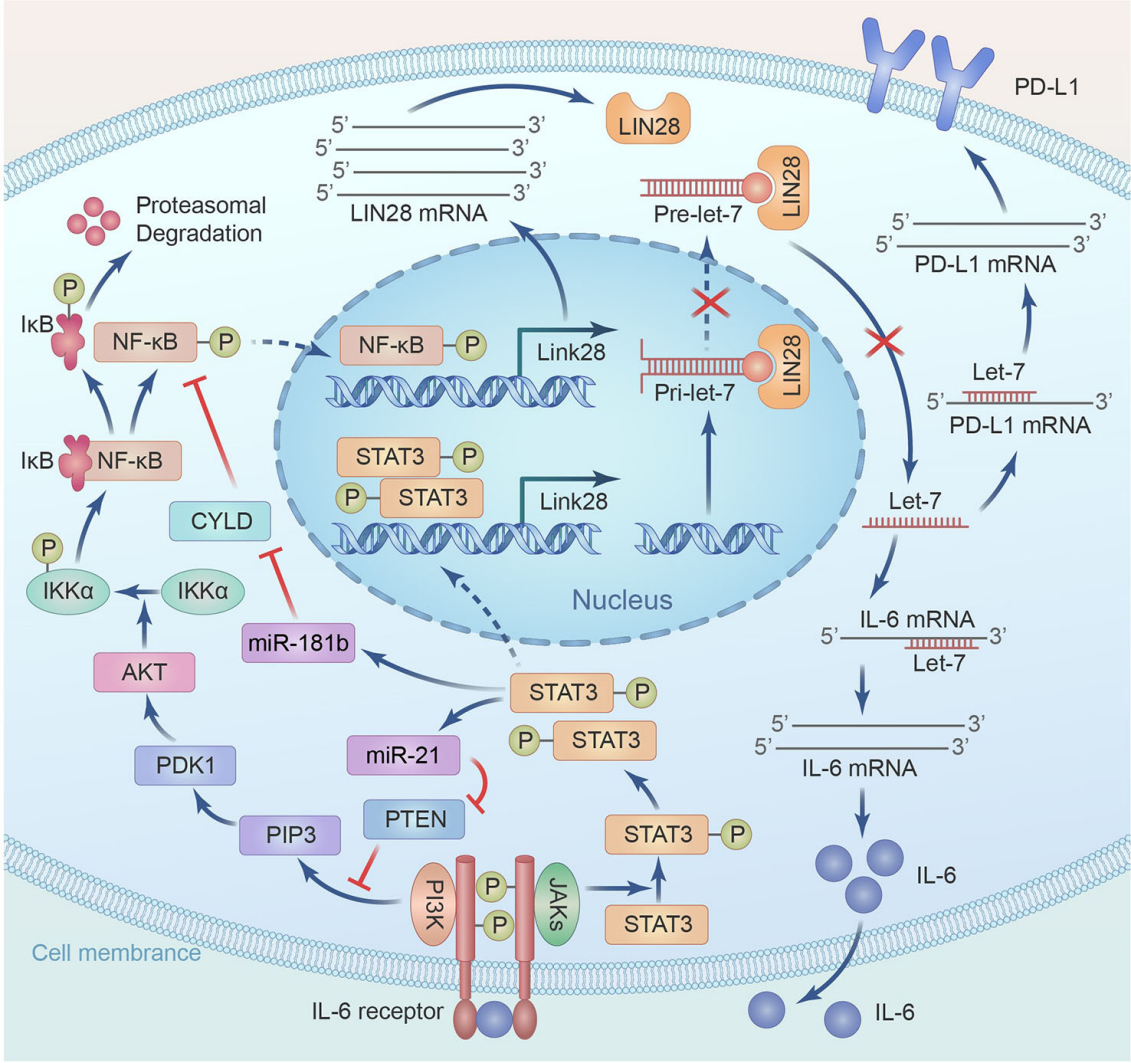
A positive feed-back between LIN28/Let7 axis and NF-κB/STAT3 mediates up-regulation of IL-6 and PD-L1.

The RNA-binding protein Lin28 is present as two homologs, Lin28A and Lin28B. Lin28A, first identified in 1997 in research on C. elegans, is commonly present in mammals (117, 118), while Lin28B was first identified in hepatocellular carcinoma; therefore, both are involved in the development and cancer (119, 120). LIN28 homologs are typically expressed in undifferentiated and pluripotent cells, and their expression is then reduced in response to development and differentiation. Let7 was first identified in C. elegans in 2001 as a typical miRNA 19-22 nucleotide in length that binds the 3’UTRs of target mRNAs, including Lin28 and PD-L1, facilitating mRNA decay and translation inhibition (121). Members of the let7 family perform vital roles in regulating cell differentiation, metabolism, and the progression of certain diseases, including tumorigenesis (122). Lin28A/B binding to pre-let-7 or pri-let-7 not only suppresses let-7 precursor processing by Dicer and Drosha into its mature form but also induces oligo-uridylation at the 3′ terminus of pre-let-7, rendering it susceptible to degradation by the 3’-5’ exonuclease Dis3L2. Thus, the Lin28-Let-7 axis is considered a double-negative feedback loop involved in regulating various biological functions; normally, most LIN28-binding sites are non-miRNA transcript, such as ribosomal and coding RNA, whose binding sequesters the LIN28 protein and competitively inhibits LIN28-repression on cellular miRNAs (123). LIN28-mediated mechanisms that are either Let-7-dependent or Let-7-independent regulate the hallmarks of cancer, such as proliferation, metabolism, the evasion of immune destruction, tumor-associated inflammation, cancer cell death, and genome instability (124) (see **Figure 3**; **Table 1**).

**Table 1:** Lin28 regulates Let7 related and Let7 independent targets in cancer.

	LIN28-Let7 axis	Let7 Independent Way
Positive feedback	STAT3 NF-kB C-Myc IL-6	Not reported
IGF signaling pathway	AKT RAS mTOR C-myc	IGF2 IGF-1R Insulin Receptor IRS2/4
Cancer Stemness		ALDH1 Nanog OCT4 SOX2
Glucose metabolism	PDK1 AKT	PDH HK1
Epithelial mesenchymal transition	HMGA2	HMGA1
Cell cycle proteins	cyclinDl/2, CDK6, CDC34, CDC25a	cyclinA/B/D, CDK1/2/4, CDC2 and CDC20
Protein Synthesis	Raptor Rictor	Not reported
RNA binding proteins		TDP-43, FUS/TLS TIA-1 hnRNP F
Histone components		Histone H2A Histone H4H Linker histone H1FX

**Figure 4:**
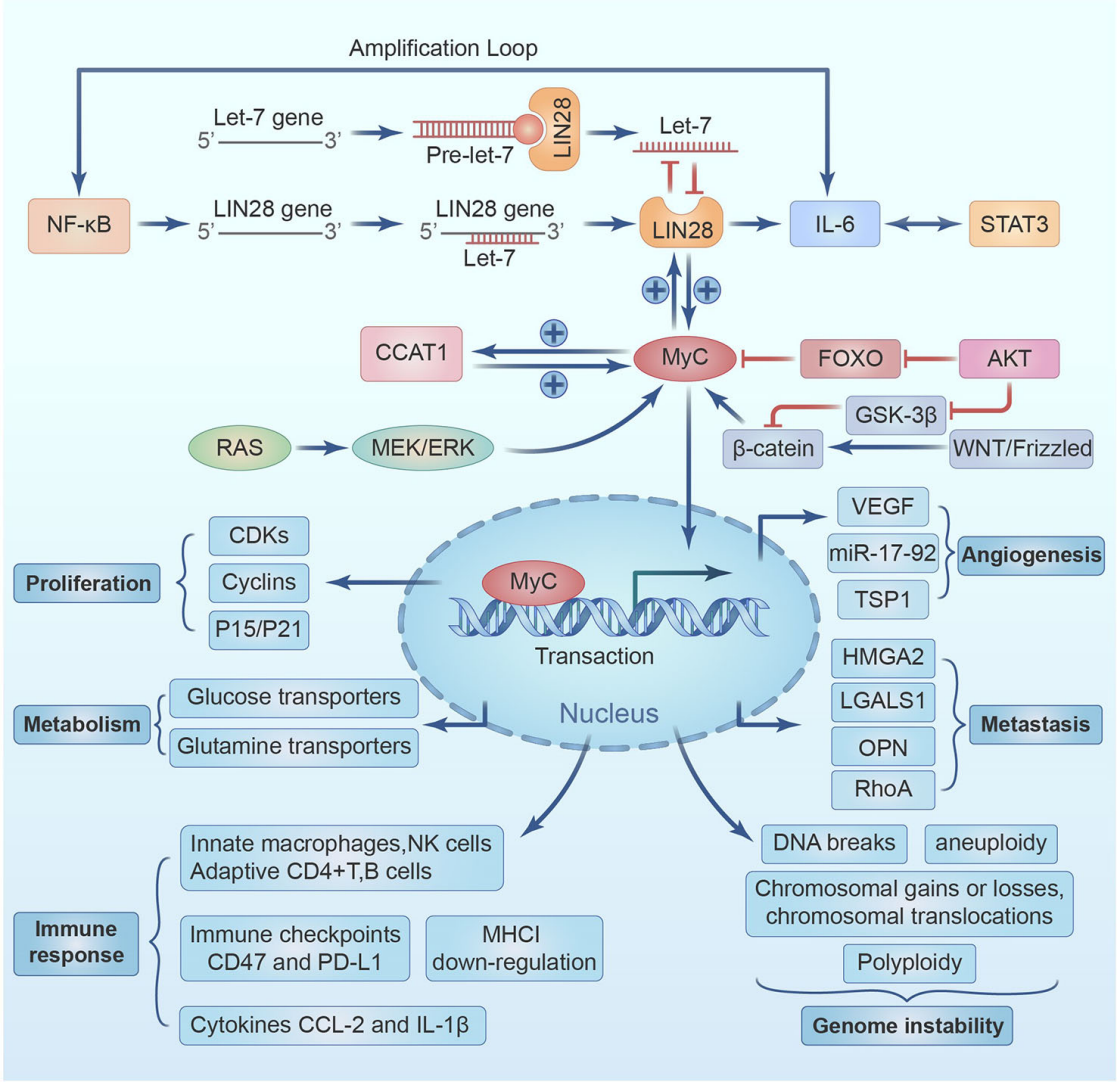
Mechanisms of Myc activation and cross-talk with the hallmarks of cancer.

Lin28 overexpression and let-7 inhibition increase IL-6 expression and subsequently activate NF-κB and STAT3, but accumulating evidence in various cancers, including HNSCC, has revealed that Lin28 overexpression and let-7 inhibition also leads to Myc overexpression. In contrast, the induction of let-7a expression significantly reduces Myc expression. The binding of Let-7 miRNA represses Myc mRNA, and Lin28 elevates Myc expression by inhibiting Let-7 miRNA binding. Moreover, Myc can increase Lin28 expression by occupying the Lin28a and Lin28b promoters, so a reciprocal positive feedback loop between Lin28 and Myc exists (124). c-Myc binds directly to the E-box component in the CCAT-1 promoter area and increases CCAT-1 expression (125). CCAT-1-L resides inside a super-enhancer region and promotes c-Myc production by facilitating chromatin looping connections among the c-Myc promoter and its enhancers *via* its interaction with CTCF (126). In addition, CCAT-1 may increase c-Myc expression by acting as a sponge for tumor-suppressor microRNAs that straightly target c-Myc (127). Therefore, a positive feedback loop among CCAT-1 and c-Myc boosts cancer cell aggressiveness by increasing the expression of each gene. Myc gene amplification is common in many solid tumor types, while chromosomal translocation is common in B-cell/T-cell leukemias and lymphomas; Myc is activated in about 70% of HNSCC *via* amplification or pathway activation (128). The detailed mechanisms of Myc activation by related signaling pathways and the multiple crosstalk mechanisms with the hallmarks of cancer are presented in **Figure 4**.

Based on mechanisms that contribute to concomitant activation, the master transcription factors NF-κB, STAT3, FOXO, AKT and Myc bind the promoter of LIN28 and PD-L1 and upregulate their expressions. Similarly, the RAS and MEK/ERK also bind with the promoter of Myc and eventually upregulate the expression of LIN28. The β-catein, GSK-3 β and WNT/Frizzled also affect the expression of Myc and LIN28. The expression of Myc leads to the activation of CDKs, Cyclins and P15/P21 which causes cell proliferation. The LIN28-Let7 axis underpins the whole IGF and PD-L1 upregulation network. Furthermore, the expression of Myc is associated with VEGF, and miR-17-92 and TSP1 that causes angiogenesis. Myc is also associated with HMGA2, LGALS1, OPN and RhoA linked with the metastasis. Let-7a downregulation is negatively linked with higher CCAT1, c-Myc, and PD-L1 expression levels in TNBC, confirming this hypothesis. Let-7a overexpression or CCAT1 inhibition dramatically suppressed PD-L1 expression in MDA-MB-231 cells and TNBC reducing cell survival and colony formation (129). Interestingly, metformin and the Lin28A inhibitor C1632 exhibited synergistic antitumor effects in oral squamous cell carcinoma *via* increasing mature Let-7 and downregulated PD-L1. In addition to the ability of metformin to decrease c-myc to induce tristetraprolin expression in breast cancer (7), it stimulates Dicer through the AMP-activated protein kinase pathways to permit the maturation of let-7 (121). Let-7 overexpression was reported to reduce PD-L1 glycosylation and endorse its deprivation in HNSCC, and the specific mechanism needs clarification (130). In addition, C1632 therapy may inhibit PD-L1 in antigen-presenting THP-1 macrophage, and the increased release of interferon and TNFα boosts T-cell-mediated anti-tumor activity (131). Despite the discovery of T helper 17 (Th17) cells in tumor tissues, their role in cancer immunity remains unknown. Mice lacking the immune cell-signaling molecule interleukin-17A (IL-17A) are more likely to develop malignant lung cancers. Instead, adoptive T-cell treatment with tumor-specific Th17 cells successfully suppressed tumor growth. Th17 cell treatment was thought to have little impact on tumor growth, dramatically stimulated the production of tumor-specific CD8+ T cells, which were crucial to the therapeutic outcome. Tumor-infiltrating dendritic cells (DCs) and CD8(+) DCs carrying tumor antigens were both enhanced in number by Th17 cells draining to lymph nodes. Th17 cells boosted the production of the chemokine CCL20 in tumor tissues. Because of this, tumor-specific CD8+ T cell activation is facilitated by the inflammation induced by Th17 cells. These results may have major consequences for anticancer immunotherapies (132). In cancer immunoediting, tumors may evade the immune system by eliminating primary histocompatibility compound class I (MHC-I) molecules. The vast majority of natural killer (NK) cells in advanced malignancies have been shown in recent research to be faulty, allowing malignant MHC-I-deficient tumors to escape the regulation of the immune system. Here, we demonstrate that a vaccination based on antigen-presenting cells (APCs) expressing tumor antigens loaded with natural killer T (NKT)-cell ligands are very effective against these late-stage tumors. They discover that MHC-I downregulation in tumors is intimately linked to developing NK-cell fatigue in tumor-bearing mice and human cancer patients. This co-expression characterizes functionally tired NK cells in advanced tumors. The anti-tumor benefits of the vaccination against advanced tumors depend partly on IL-21’s ability to restore NK-cell activity. These findings provide light on the mechanism behind the generation of NK-cell dysfunction in advanced tumors and may help guide the design of immunotherapeutic approaches to this disease. The methodologies for studying linked biomarkers, like high-throughput sequencing of T-cell receptors (TCRs), are discussed, as are the bioinformatics involved in designing personalized, neoantigen-based vaccines, including detection of mutations and the prediction of potential epitopes, as well as data analysis and the bioinformatics quantitative of immune cell infiltration in tumor tissues. (133).

## 6 Conclusions

These findings show that mutually connected IL-6, EGFR, and LIN28/Let7 axis-related mechanisms modulate IGF and PD-L1 upregulation in the HNSCC microenvironment, which comprehensively influences immunity, inflammation, metabolism, and metastasis and might be fundamental for overcoming therapy resistance. IGF-1/2 gene expression is stimulated by IL-6, which also cross-activates the IGF-1R signaling pathway, NF-κB, and STAT3 genes to promote their expression. PD-L1 is stabilized and transported to the cell surface through STT3-mediated PD-L1 glycosylation, which is facilitated by IL-6/JAK1. It has been concluded that PD-L1 and IGF overexpression in HNSCC are mediated by IL-6, EGFR, and LIN28/Let7-related pathways. This extensively affects immunity, inflammation, metabolism, and metastasis in the tumor microenvironment and may be crucial for overcoming therapeutic resistance.

## Author contributions

WL proposed the idea. SF and HY affirmed its value. WL developed the train of thought and wrote the article. JL, XJ and YX took part in the discussion. SF revised the article. All authors contributed to the article and approved the submitted version.

## Glossary

**Table:**

ADCC	antibody-dependent cell-mediated cytotoxicity
ADAM	a disintegrin and metalloproteinase domain-containing protein
AP-1	activator protein 1
AKT	protein kinase B
C/EBPβ	CCAAT/enhancer-binding protein beta
CCL	C- C motif chemokine
CNTF	ciliary neurotrophic factor
CTCF	CCCTC-Binding Factor
CXCR1/2	Chemokine C-X-C Receptor ½
DAG	Diacylglycerol
EGFR	epidermal growth factor receptor
ERK	extracellular signal-regulated kinase
EMT	Epithelial- Mesenchymal Transition
FOXO	forkhead box O
GM-CSF	granulocyte macrophage colony-stimulating factor
GPCRs	G protein coupled receptors
GSK3	Glycogen synthase kinase-3
HNSCC	head and neck squamous cell carcinomas
IGFBP	insulin-growth factor binding proteins
IGFR	insulin-like growth factor receptor
HER	human epidermal growth factor receptor
IGF	insulin-like growth factor
IKKα	IkappaB kinase alpha
IL-6	Interleukin-6
IP3	and inositol 1,4,5-tri-sphosphate
IRS-1	insulin receptor substrates 1
JAK	Janus kinase
LIF	leukemia inhibitory factor
MCP	monocyte chemoattractant protein
MDM2	mouse double minute 2
MDSC	myeloid-derived suppressor cells
MEK	mitogen-activated protein kinase
NF-κB	Nuclear Factor Kappa B
mTOR	Mammalian target of rapamycin
OSM	oncostatin M
PDK1	phosphoinositide-dependent protein kinase1
PI3K	phosphatidylinositol 3-kinase
PD-L1	programmed death-ligand 1
PIAS	Phospprotein inhibitors of activated STATs
PIP2	Phosphatidylinositol 4,5-bisphosphate
PIP3	Phosphatidylinosito 3,4,5-trisphosphate
PKM2	Pyruvate kinase M2
PDLIM2	PDZ-LIM domain-containing protein 2
PP2A	protein phosphatase 2A
PTEN	phosphatase and tensin homologue deleted on chromosome 10
PTPRs	protein tyrosine phosphatase receptors
Raf	rapidly accelerated fibrosarcoma
RAS	rat sarcoma
RTKs	receptor tyrosine kinases
SHP	SH2 containing tyrosine phosphatases
SOCS	suppressor of cytokine signaling proteins
SOX2	SRY-box 2
SREBP	sterol response element binding protein
STAT3	signal transducer and activator of transcription 3
TKI	tyrosine kinase inhibitors
TLRs	toll-like receptors
TNBC	Triple negative breast cancer
TP63	tumor protein p63
TSC	Tuberous Sclerosis Complex
USPs	Ubiquitin-specific proteases
VEGFR	vascular endothelial growth factor receptors
VEGF	vascular endothelial growth factors
XIAP	X-linked inhibitor of apoptosis protein
